# Density Functional Theory Study on Na^+^ and K^+^ Catalysis in the Transformation of Glucose to Fructose and HMF in Hydrothermal Environments

**DOI:** 10.3390/molecules29204849

**Published:** 2024-10-13

**Authors:** Long Gao, Qihao Chen, Yanhong Wang, Deyong Che, Baizhong Sun, Shuai Guo

**Affiliations:** School of Energy and Power Engineering, Northeast Electric Power University, Jilin 132012, China; gaolong0607@163.com (L.G.); 19861834570@163.com (Q.C.); chedeyong163@163.com (D.C.); sunbaizhong@126.com (B.S.)

**Keywords:** density functional theory, catalytic hydrothermal carbonization, glucose, soluble polymer

## Abstract

Hydrothermal carbonization (HTC) is an efficient method for converting biomass into biochar. Hydrochar contains catalytic components such as alkali and alkaline earth metals (AAEMs); however, the mechanisms by which highly active metals such as potassium (K) and sodium (Na) catalyze the conversion of small carbon–water compounds into hydrochar in hydrothermal environments remain unclear. In this study, glucose was used as a small molecule model, and Na^+^ and K^+^ were used as catalysts to investigate the catalytic reaction mechanism during the hydrothermal process using density functional theory (DFT). In the presence of different ions at various binding sites, glucose isomerizes into fructose, which subsequently undergoes three consecutive dehydration reactions to form 5-hydroxymethylfurfural (HMF). The results indicate that the catalytic effectiveness of Na^+^ and K^+^ in the isomerization of glucose to fructose is optimal when interacting with specific oxygen sites on glucose. For Na^+^, the interaction with the O1 and O2 oxygens provides the lowest reaction barrier of 37.16 kcal/mol. For K^+^, the most effective interactions are with the O3 and O4 oxygens and the O5 and O6 oxygens, resulting in reduced reaction barriers of 54.35 and 31.50 kcal/mol, respectively. Dehydration of fructose to HMF catalyzed by Na^+^ ions, the catalytic effectiveness at different positions is ranked as O5O6 > O1O5, whereas for K^+^, the ranking is O1O5 > O5O6. This study explores the catalytic effects of Na^+^ and K^+^ at different binding sites on the hydrothermal reactions of glucose at the atomic level, offering theoretical support for designing catalysts for the HTC of sludge.

## 1. Introduction

**Figure 1:**
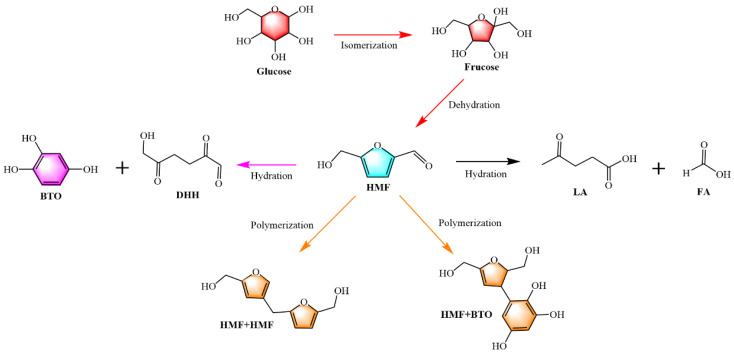
Reaction mechanism for the transformation of glucose into hydrochar precursor polymers.

With the acceleration of urbanization, the domestic water consumption of the population has been increasing annually [1]. Consequently, the volume of wastewater requiring treatment has also been rising each year. Traditional methods for treating sewage sludge are costly, flawed, and have low resource utilization rates [2]. Therefore, the treatment of sewage sludge has become an urgent issue that needs to be addressed [3]. HTC is a thermochemical process [4] that converts biomass into harmless hydrochar by reacting continuously for several hours at temperatures ranging from 150–300 °C under self-generated pressure [5], thereby effectively enhancing the treatment efficiency of sewage sludge. Several important intermediates, such as glucose, HMF, and fructose, are produced during the HTC process. These intermediates can be further converted into various derivatives with diverse applications [6]. According to Figure 1, in a hydrothermal environment, organic macromolecules hydrolyze into their corresponding monomers. For example, polysaccharides hydrolyze into glucose, and proteins hydrolyze into amino acids. Initially, glucose is isomerized into fructose, which then undergoes consecutive dehydration reactions to form HMF. HMF reacts with water in a hydration reaction to produce a series of small organic molecules. These small organic molecules undergo polymerization reactions to form hydrochar precursor polymers. Finally, the hydrochar precursor polymers are polymerized further to produce clean fuel hydrochar.

At the microscopic level, in the field of DFT research, DFT has been extensively used to explore hydrolysis mechanisms and the formation of related products, supporting experimental inferences and validations. Hu et al. [7] employed quantum chemical calculations to elucidate the mechanism of HMF production during glucose pyrolysis, revealing that glucose preferentially forms fructose through a ring-opening reaction, followed by the dehydration of fructose to form HMF. Building on this, Wei et al. [8] explored the mechanism of glucose catalysis forming levulinic acid (LA) in aqueous solutions, demonstrating that LA is primarily formed through the hydration reaction of HMF. Zhang et al. [9] reviewed the progress in the conversion of glucose to HMF and LA, discussing the effects of catalysts and solvent systems. They particularly emphasized the mechanism of glucose dehydration. Jyotsna S. Arora et al. [10] investigated the reaction mechanisms of glucose decomposition in the presence and absence of alkali and alkaline earth metal ions using a combination of pyrolysis experiments and DFT calculations. The DFT results indicated that Na and K ions catalyze the formation of glucose, while Mg and Ca ions exhibit anti-catalytic effects. However, the catalytic effects of Na and K ions on glucose in hydrothermal environments have not yet been studied. Guo et al. [11] discovered through the study of reaction mechanisms that the AlCl_4_^-^ anion facilitates the isomerization of glucose to fructose, while the -SO_3_H group can form hydrogen bonds with fructose, promoting the dehydration reaction. In significant research on hydrothermal environments, Guo et al. [12] used DFT calculations to study the catalytic role of water molecules under defined hydrothermal conditions. Their work deepens our understanding of the HTC process and paves the way for more effective conversion of these resources into value-added chemicals and fuels.

At the microscopic level, in the study of different ions in the field of HTC, Abdullayev et al. [13] used glucose as a model compound to explore the catalytic activity of Brønsted acidic ionic liquids in this conversion. Regarding the catalysts involved in the HTC process, Tian et al. [14] demonstrated that the Lewis acid SnCl_4_ effectively catalyzes the conversion of glucose to 5-HMF. Similarly, Moliner et al. [15] demonstrated that large-pore zeolites containing Sn-Beta can efficiently and selectively isomerize glucose to fructose in aqueous solutions. Furthermore, Zhang et al. [16] conducted a kinetic study on the conversion of glucose catalyzed by CrCl_3_^−^ to HMF in an ionic liquid. They found that although Cl^−^ catalyzes the isomerization reaction, its activity is significantly inhibited by hydroxyl hydrogen bonding. Pyo et al. [17] revealed that LA is a promising organic biorefinery platform chemical and an intermediate for the synthesis of fuels, chemicals, and polymers. Li et al. [18] discovered that Brønsted acids can catalyze the production of HMF from fructose. Parveen et al. [19] revealed that alkaline functional groups in organic catalysts isomerize glucose to fructose, while Brønsted acidic groups catalyze the dehydration of fructose to HMF. Yang et al. [20] utilized glucose as a feedstock to prepare HMF in different organic solvents catalyzed by CrCl_3_ and Brønsted acids. Guo et al. [21] used glucose as a small molecule model and employed DFT to study the reaction mechanism during sludge hydrothermal processes, with Lewis acid (CuCl_2_) and Brønsted acid (NH_4_^+^) as catalysts. Glucose is isomerized to fructose under the catalysis of CuCl_2_ and Cl^−^, while fructose undergoes consecutive dehydration reactions to generate HMF under the catalysis of NH_4_^+^. HMF undergoes multiple hydration and dehydration reactions catalyzed by NH_4_^+^ to produce small organic molecules, which polymerize to form hydrochar precursor polymers. In the study of multi-catalytic active sites for the isomerization of glucose to fructose [22], the catalytic activity was first investigated by configuring different proportions of catalysts in cellulose-derived biochar. The results indicate that the main active sites of the catalyst can provide weakly acidic and weakly basic sites, avoiding excessive attacks on glucose by strong acidic and strong basic sites. The DFT and wave function analysis results indicate that MgO has a significant impact on the generation of acyclic glucose in open-loop reactions, while Al (OH)^3+^ has a significant impact on promoting the hydrogen transfer isomerization of acyclic glucose to fructose. This study demonstrates the feasibility of synthesizing highly efficient catalysts with multiple active sites through simple hydrothermal carbonization and further calcination.

Currently, research on the hydrothermal catalytic conversion of sludge to hydrochar is primarily experimental, and there is insufficient investigation at the atomic level regarding the binding modes of Lewis acids with glucose and their catalytic effects at the atomic level. Therefore, this study selects Na^+^ and K^+^ as catalysts for the conversion of glucose to HMF to elucidate the reaction mechanism and energy barrier changes during the hydrothermal process. DFT is employed to explore the mechanism of sludge carbohydrate hydrolysis to form hydrochar precursor polymers, providing theoretical guidance for further experimental research. By comparing reaction pathways and their energy barriers, the optimal binding modes and catalytic effects of Na ions and K ions with glucose and fructose are determined.

## 2. Results and Discussion

### 2.1. Mechanistic Study of Glucose-to-Fructose Conversion Catalyzed by K^+^ and Na^+^ Ions

**Figure 2:**
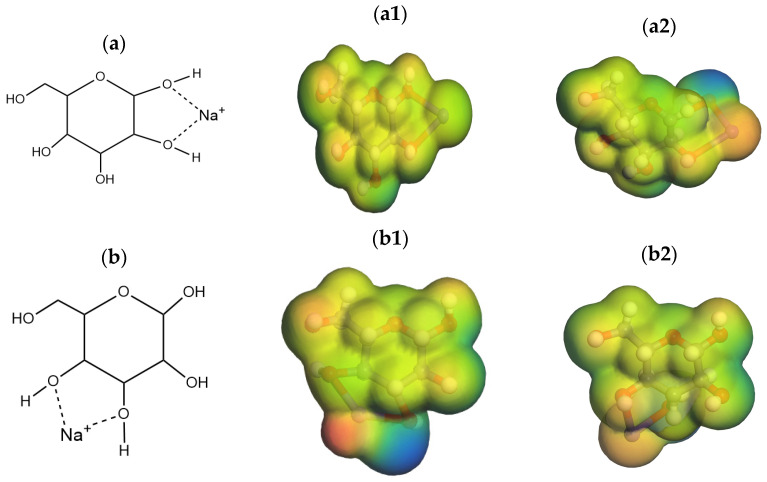

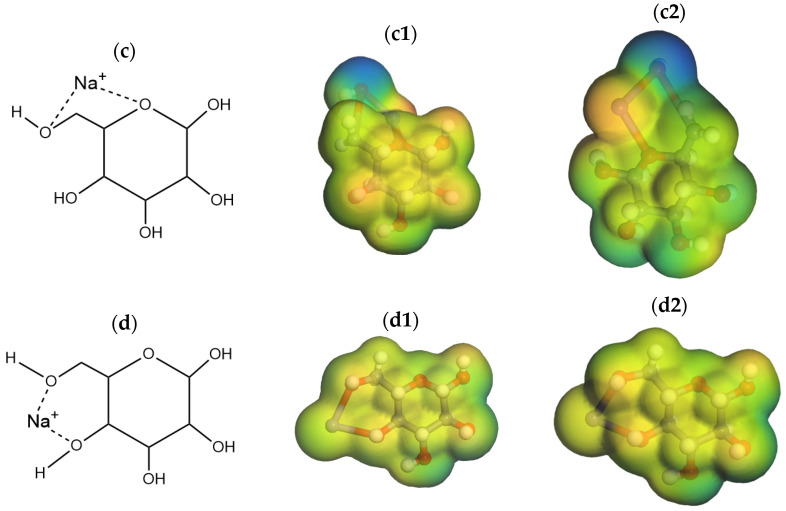
Four binding modes of Na ion with glucose: (**a**) O1, O2; (**b**) O3, O4; (**c**) O5, O6; (**d**) O4, O6; (**a1**–**d1**) the electrostatic surface of Na ions; (**a2**–**d2**) the electrostatic surface of K ions.

In hydrothermal reactions, ion coordination typically occurs at specific functional groups within the reaction system. These functional groups mainly include oxygen-containing groups such as hydroxyl, aldehyde, and ketone groups. These groups provide coordination sites that can form complexes with ions, thereby influencing the progress of the reaction. In glucose molecules, there are multiple functional groups that can serve as coordination sites for ions, primarily hydroxyl groups. According to Figure 2, the position where glucose molecules are most easily coordinated can be obtained. During hydrothermal reactions, K^+^ or Na^+^ ions may form coordination bonds with the oxygen atoms of these functional groups. Glucose molecules contain several hydroxyl functional groups, and each hydroxyl oxygen atom is a potential coordination site. Different ions may form coordination bonds with one or more of these hydroxyl oxygen atoms. Glucose molecules can coordinate with sodium ions through their hydroxyl (OH) groups. The positive charge of the sodium ion is electrostatically attracted to the oxygen atoms of the glucose hydroxyl groups. This coordination can occur repeatedly, forming one or more complexes between sodium ions and glucose molecules. Specifically, glucose molecules have multiple hydroxyl functional groups available for coordination.

According to previous research by Jyotsna S. Arora et al. [10], Lewis acids play a significant role in the isomerization of glucose to fructose. In this process, glucose undergoes ring-opening, hydrogen transfer, and ring-closing reactions. The coordination of Na^+^/K^+^ ions may reduce the reaction barrier by affecting the electronic density distribution of the reactants. Specifically, when the Na^+^/K^+^ forms coordinate with the hydroxyl group, they can alter the local electronic density through electrostatic attraction and charge transfer effects, thereby increasing the nucleophilicity of the reaction site. This change facilitates the migration of hydrogen atoms in the transition state, leading to a lowered reaction barrier. The presence of Na^+^/K^+^ may weaken the strength of the OH bond. Coordinated ions can reduce the dissociation energy of the OH bond through the formation of hydrogen bonds or electrostatic interactions, thereby decreasing the energy required for hydrogen atom transfer. This study selects Na^+^ and K^+^ as representatives of Lewis acids, as these ions readily form stable complexes with the hydroxyl groups in glucose. As illustrated in Figure 2, there are four binding modes between Na^+^ ions and glucose: (1) O1, O2; (2) O3, O4; (3) O5, O6; (4) O4, O6. Among these, the hydroxyl groups at positions C1 and C2 are more likely to form complexes with sodium ions. Additionally, the hydroxyl groups at positions C3, C4, and C6 can also participate in coordination, though their roles are relatively weaker. The different ways in which glucose molecules bind to Na and K ions at different positions are shown in Figure 2 and are represented by electrostatic potential diagrams.

Analysis from previous literature [10] revealed four possible binding sites for Na^+^ ions, namely O1O2, O3O4, O4O6, and O5O6, while K^+^ ions may bind to two sites, O3O4 and O5O6, when coordinating with glucose molecules. By analyzing the electrostatic potential maps in Figure 2, the distribution of charges within the molecules and on their surfaces can be observed, which is crucial for predicting intermolecular electrostatic interactions and their roles in catalytic reactions. Particularly, regions of lower electrostatic potential at electronegative centers on the molecular surface suggest these areas might serve as reactive sites. Furthermore, Fukui function analysis was performed to evaluate the *f*− values for different positions of K^+^ ions, including positions O1O2 and O4O6. Specifically, regions with high values of the Fukui function *f*− indicate stronger nucleophilicity and potential participation as electron donors in reactions. However, the Fukui function values for K^+^ ions at positions O1O2 and O4O6 are noticeably higher or lower than those at other positions.

#### 2.1.1. Mechanistic Analysis of Four Distinct Binding Modes of Na^+^ Ion with Glucose

**Figure 3:**
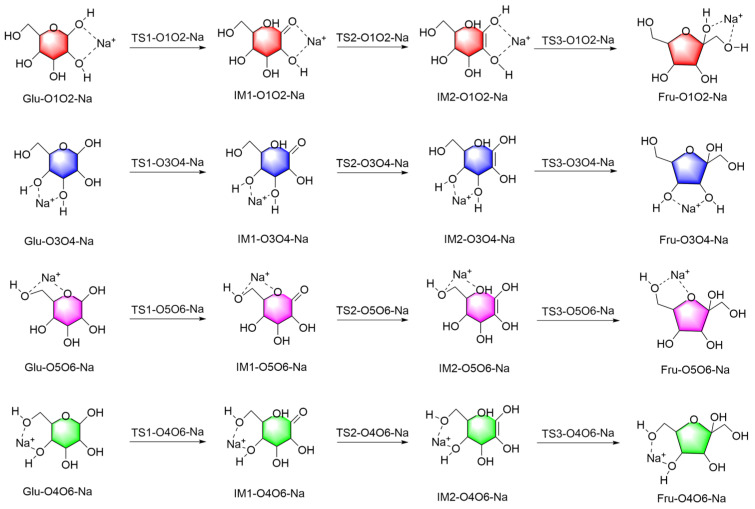
Reaction mechanism diagrams for four distinct binding modes of Na^+^ with glucose.

The hydroxyl hydrogen atom at the C1 position of the glucose molecule transfers to the O5 position, forming a hydroxyl group. This process, facilitated by the transition state TS1, results in the ring opening and the formation of the intermediate IM1. This step requires overcoming a relatively high energy barrier due to the need to break the original ring structure of the glucose molecule, which involves a significant energy cost. Subsequently, the hydrogen atom at the C2 position in intermediate IM1 transfers to the O1 position, passing through the transition state TS2 to form a hydroxyl group and generate intermediate IM2. Finally, in intermediate IM2, the hydrogen atom of the hydroxyl group at O5 transfers to the C2 position, transforming the molecule from a linear structure to the stable five-membered ring structure of fructose. The above describes the reaction mechanism for the conversion of glucose to fructose. On this basis, Figure 3 presents the mechanistic study of the four different binding modes of Na^+^ with glucose. The reaction process can be divided into two main stages. In the first stage, the complex formed by glucose and Na^+^ (Glu-Na) undergoes a ring-opening reaction, specifically manifested as the transfer of the hydrogen atom on O1 to the O5 position, forming the intermediate IM1-Na. This ring-opening process requires overcoming a high energy barrier because breaking the stable cyclic structure of the glucose molecule demands substantial energy. However, the catalytic effect of Na^+^ facilitates this process by lowering the bond dissociation energy of the C1-O5 bond, allowing the reaction to proceed smoothly. In the second stage, the conversion from IM2-Na to Fru-Na involves two key hydrogen atom transfers. First, the hydrogen atom at the C2 position transfers to the hydroxyl group at the C1 position, forming the intermediate IM2-Na. This process is successful because the presence of Na^+^ significantly reduces the energy barrier of the transition state TS2, greatly enhancing the reaction’s efficiency. Next, the hydrogen atom from the hydroxyl group at the C5 position transfers to the C2 position, ultimately forming the stable fructose-Na complex (Fru-Na). This step further reduces the bond energy of the C2-O bond, allowing the reaction system to transition from a high-energy intermediate to a more stable product, thereby completing the entire conversion process.

The reaction energy barriers for four different binding modes of Na^+^ with glucose indicate that Na^+^ binds to glucose molecules at different positions, proceeding primarily through a two-stage reaction. These four positions are described in the following order: O1O2, O3O4, O5O6, and O4O6. In the first stage, the Glu-Na complex undergoes a ring-opening reaction, with the hydrogen atom on O1 transferring to O5 to form IM1-Na. The reaction energy barriers for the four different positions are 8.24, 14.13, 35.14, and 30.28 kcal/mol, respectively. Compared to the non-catalytic pathway from Glu to IM1, these energy barriers are reduced by 37.16, 31.27, 10.26, and 15.11 kcal/mol, respectively. By comparing the reduction in reaction energy barriers, the catalytic effectiveness from best to worst in this process is O1O2 > O3O4 > O4O6 > O5O6.

In the second stage, the process from IM2-Na to Fru-Na involves two hydrogen transfers: (1) the hydrogen at the C2 position transfers to the hydroxyl group at C1, and (2) the hydrogen from the hydroxyl group at C5 transfers to C2. For the first hydrogen transfer, the transition from IM1-Na to IM2-Na has reaction energy barriers of 30.67, 53.38, 38.69, and 46.73 kcal/mol for the four different positions, respectively. Compared to the non-catalytic pathway from IM2 to IM3, the energy barriers are reduced by 48.44, 25.73, 40.42, and 32.38 kcal/mol, respectively. By comparing the reductions in reaction energy barriers, the catalytic effectiveness in this process from best to worst is O1O2 > O5O6 > O4O6 > O3O4. For the second hydrogen transfer, the transition from IM2-Na to Fru-Na has reaction energy barriers of 15.43, 8.19, 37.50, and 11.02 kcal/mol for the four different positions, respectively. Compared to the non-catalytic pathway from IM2 to Fru, the energy barriers are reduced by 21.88, 29.12, −0.18, and 26.29 kcal/mol, respectively. By comparing the reductions in reaction energy barriers, the catalytic effectiveness in this process from best to worst is O3O4 > O4O6 > O1O2 > O5O6.

**Figure 4:**
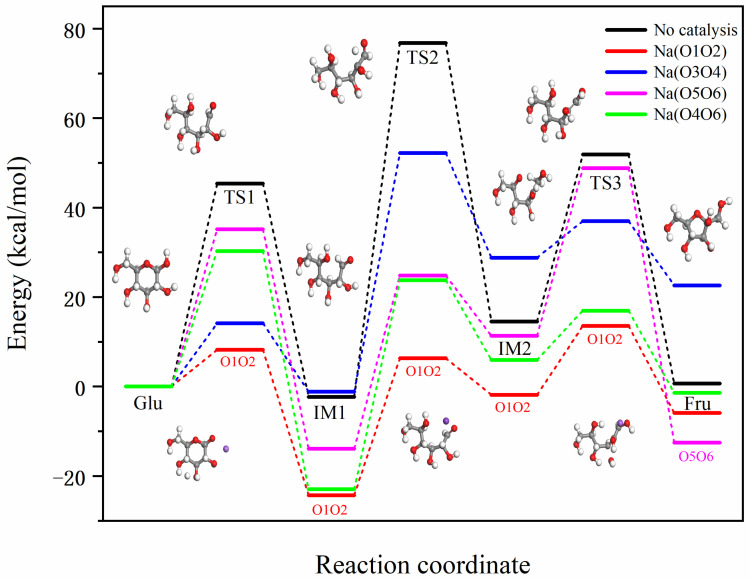
Reaction energy barrier diagrams for four distinct binding modes of Na^+^ with glucose.

In the entire reaction process at the O1O2 position, the highest and lowest reaction energy barriers are 13.56 kcal/mol and −24.31 kcal/mol, respectively, with an overall reaction energy barrier of 37.88 kcal/mol. At the O3O4 position, the highest and lowest reaction energy barriers are 52.20 kcal/mol and −1.18 kcal/mol, respectively, with an overall reaction energy barrier of 53.38 kcal/mol. At the O5O6 position, the highest and lowest reaction energy barriers are 48.82 kcal/mol and −13.87 kcal/mol, respectively, with an overall reaction energy barrier of 62.70 kcal/mol. At the O4O6 position, the highest and lowest reaction energy barriers are 30.28 kcal/mol and −22.97 kcal/mol, respectively, with an overall reaction energy barrier of 53.25 kcal/mol. In comparison, the non-catalyzed pathway has an overall reaction energy barrier of 79.11 kcal/mol. Therefore, the reductions in the overall reaction energy barriers are 41.23, 25.73, 16.41, and 25.85 kcal/mol for the O1O2, O3O4, O5O6, and O4O6 positions, respectively. Considering the three stages and the overall reaction energy barriers, the comprehensive evaluation of the catalytic effectiveness of Na^+^ at different positions is as follows: O1O2 > O4O6 > O3O4 > O5O6. The Na^+^ ion [10] stabilizes the coordination complexes by binding to glucose atoms, thus providing stability to the high-energy transition states of the reaction. However, the binding sites of Na^+^ ions in the transition state may differ from those in the ground-state complex. The above content is a detailed description of the catalytic effect of Na^+^ at different positions in Figure 4.

#### 2.1.2. Mechanistic Analysis of Two Different Binding Modes of K^+^ Ion with Glucose

**Figure 5:**
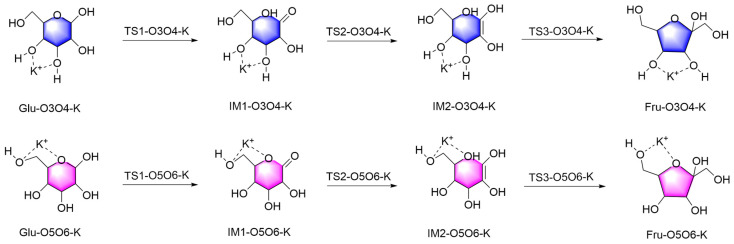
Reaction mechanism diagrams for two different binding modes of K^+^ with glucose.

Figure 5 illustrates the reaction mechanism of glucose catalyzed by K^+^ at two different binding sites. Previous analyses determined that the optimal binding positions for K^+^ on the glucose molecule are O3O4 and O5O6. Thus, the conversion of glucose to fructose is divided into two stages. In the first stage, Glu-K undergoes a ring-opening reaction, where the H atom at O1 transfers to O5, forming IM1-K. The reaction energy barriers for the two binding positions are 18.16 and 28.36 kcal/mol, respectively. Compared to the non-catalyzed pathway from Glu to IM2, the energy barriers are reduced by 27.24 and 17.04 kcal/mol, respectively. This comparison indicates that the catalytic effectiveness in this process follows the order: O3O4 > O5O6.

In the second stage, the conversion from IM1-K to Fru-K involves two hydrogen transfers: (1) the transfer of the hydrogen atom from the C2 position to the hydroxyl group at the C1 position and (2) the transfer of the hydrogen atom in the hydroxyl group at the C5 position to the C2 position. For the first hydrogen transfer from IM1-K to IM2-K, the reaction energy barriers for the two binding sites are 24.76 and 36.75 kcal/mol, respectively. Compared to the non-catalyzed pathway from IM2 to IM3, the energy barriers are reduced by 54.35 and 42.36 kcal/mol, respectively. This comparison indicates that the catalytic effectiveness in this process follows the order: O3O4 > O5O6. For the second hydrogen transfer from IM2-K to Fru-K, the reaction energy barriers for the two binding sites are 18.32 and 5.81 kcal/mol, respectively. Compared to the non-catalyzed pathway from IM3 to Fru, the energy barriers are reduced by 18.99 and 31.50 kcal/mol, respectively. This comparison indicates that the catalytic effectiveness in this process follows the order: O5O6 > O3O4.

**Figure 6:**
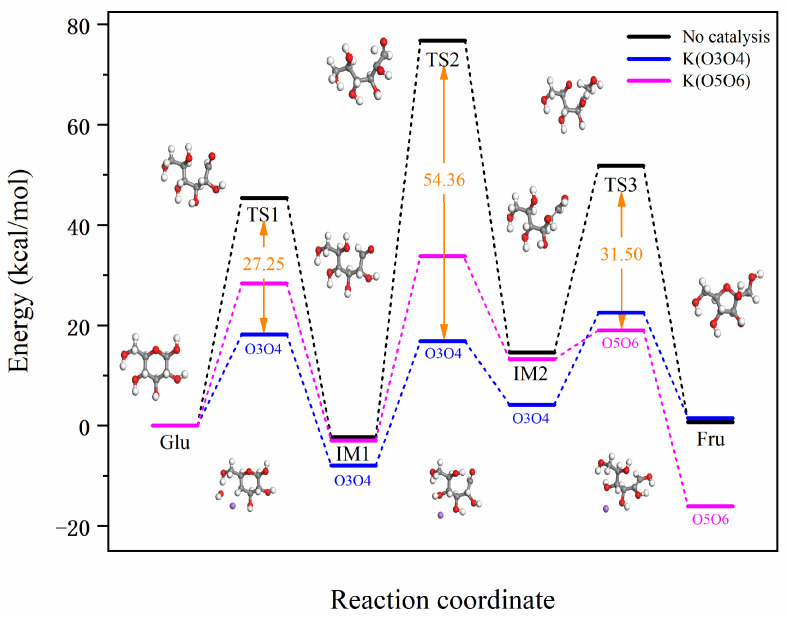
Reaction energy barrier diagrams for two different binding modes of K^+^ with glucose.

For a complex reaction system such as a catalytic cycle, the reaction energy barrier of the entire process refers to a series of energy barriers that need to be overcome throughout the entire cycle, starting from the initial substrate through the participation of the catalyst and various intermediate states, until the final product is generated and the catalyst is restored. Based on Figure 5, which illustrates the reaction energy barriers for the two different binding modes of K^+^ with glucose, it can be observed that for the O3O4 position, the highest and lowest points of the reaction energy barrier throughout the entire reaction are 22.50 and −7.97 kcal/mol, respectively, with an overall reaction energy barrier of 30.47 kcal/mol. For the O5O6 position, the highest and lowest points of the reaction energy barrier are 33.79 and −16.08 kcal/mol, respectively, with an overall reaction energy barrier of 49.87 kcal/mol. In contrast, the overall reaction energy barrier without catalysis is 79.11 kcal/mol. Compared to the non-catalyzed reaction, the overall reaction energy barriers are reduced by 48.64 and 29.24 kcal/mol for the O3O4 and O5O6 positions, respectively. Considering the reaction energy barriers at these three stages and the overall process, the catalytic efficiency of K^+^ at different positions is evaluated as follows: O3O4 > O5O6. The above content is a detailed description of the catalytic effect of K^+^ at different positions in Figure 6.

**Figure 7:**
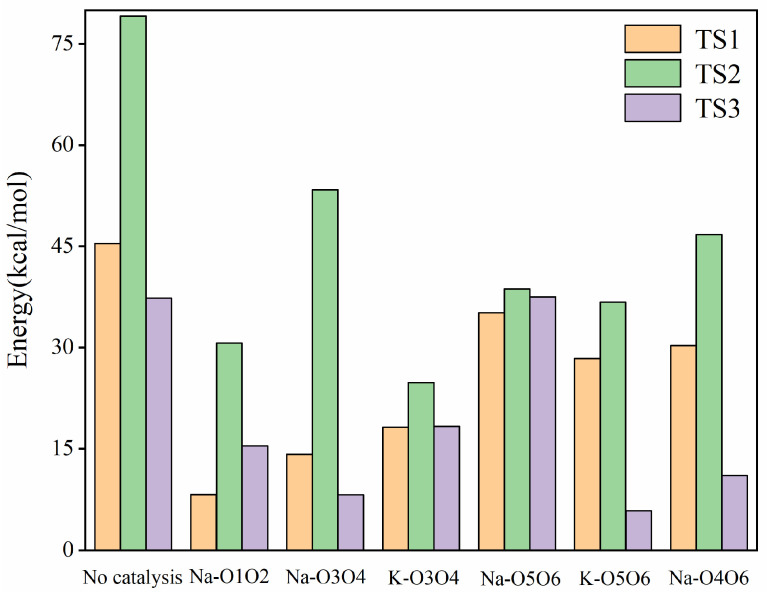
Bar graph of reaction energy barriers for Na^+^ and K^+^ catalyzed Glu-Fru reaction.

**Figure 8:**
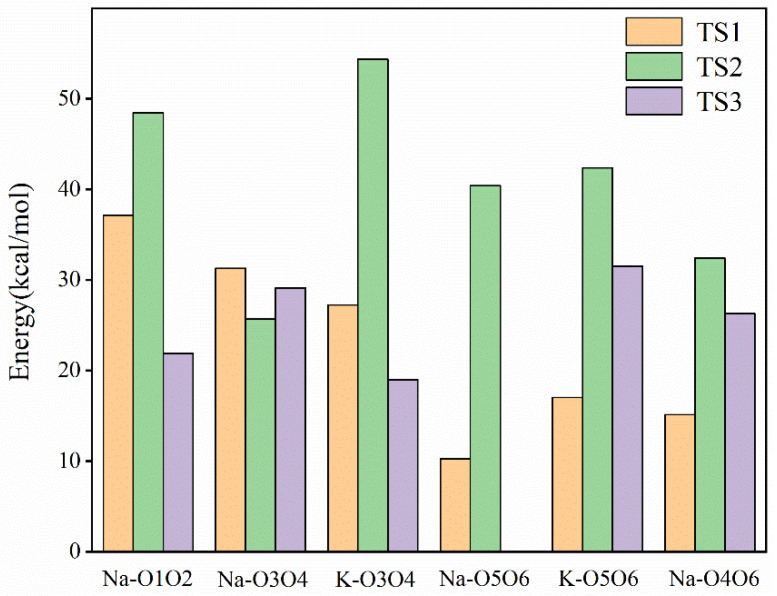
Bar graph of reduced energy barriers for Na^+^ and K^+^ catalyzed Glu-Fru reaction.

Figure 7 shows the actual energy barriers for Na^+^ and K^+^ catalyzed Glu-Fru reactions. Figure 8 illustrates how these barriers are lowered by the catalysts at different stages. The bar graph of reduced reaction energy barriers for Na^+^ and K^+^ catalyzed Glu-Fru, depicted in Figure 8, allows for a comparison of the catalytic effects of different ion positions across the three stages. In the TS1 reaction stage, Na-O1O2 has the best overall catalytic effect, reducing the energy barrier by 37.16 kcal/mol. In the TS2 reaction stage, K-O3O4 exhibits the best overall catalytic effect, with a reduction in the reaction energy barrier of 54.35 kcal/mol. In the TS3 stage, Na-O3O4 reduces the reaction energy barrier by 29.11 kcal/mol, whereas K-O5O6 reduces it by 31.50 kcal/mol, making K-O5O6 the most effective catalyst in the third stage.

### 2.2. Mechanistic Study of Fructose to HMF Conversion Catalyzed by Na and K Ions

**Figure 9:**
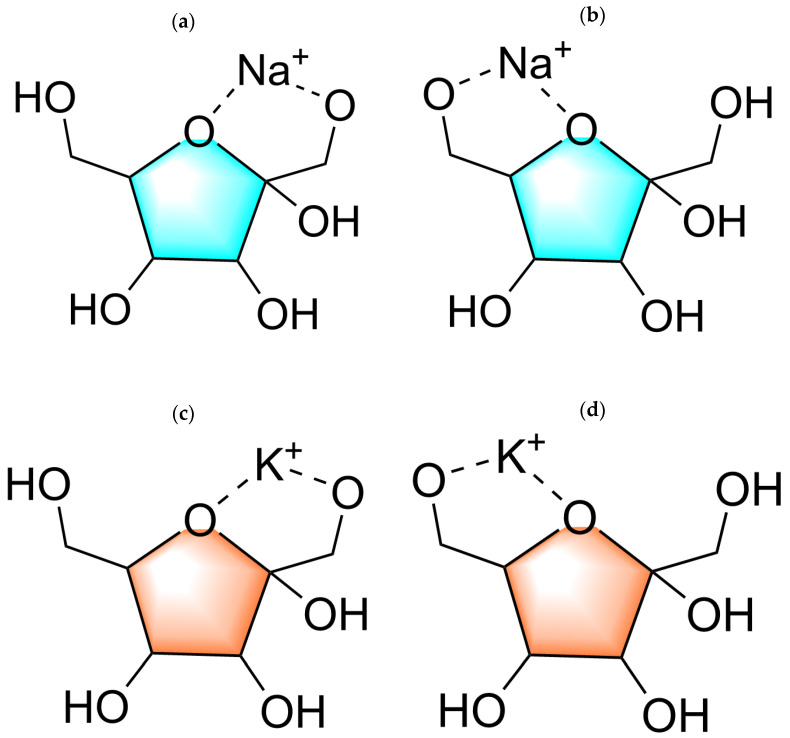
Two binding modes of Na Ion with fructose (**a**) O1, O5; (**b**) O5, O6 and two binding modes of K ion with fructose (**c**) O1, O5; (**d**) O5, O6.

During the conversion of fructose to HMF, the dehydration reaction typically involves the loss of hydroxyl and aldehyde groups, making these functional groups potential ion coordination sites. Different ions, such as Na^+^ or K^+^, can form coordination bonds with the oxygen atoms in these functional groups and stabilize intermediates. Specifically, possible coordination sites include the hydroxyl oxygen, aldehyde oxygen in the fructose molecule, and the ketone oxygen that may form in intermediates. Introducing K^+^ or Na^+^ ions into the hydrothermal process can impact the conversion of fructose to HMF in various ways, influencing the reaction selectivity, rate, and product distribution. This coordination reaction may involve the ions coordinating with functional groups in the fructose molecule and affecting overall reaction kinetics by stabilizing intermediates or altering reaction pathways. This study selects Na^+^ and K^+^ as representatives of Lewis acids, as these ions readily combine with the hydroxyl groups in fructose to form stable complex structures. According to Figure 9, there are two coordination modes: (1) O1, O5; (2) O5, O6.

**Figure 10:**
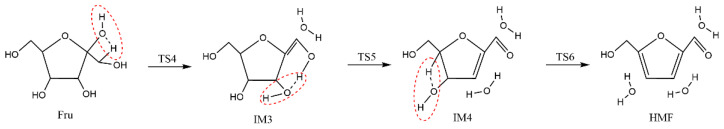
Reaction pathway diagram for fructose dehydration to form HMF.

Figure 10 illustrates the reaction pathway for the dehydration of fructose to form HMF. Hu et al. [7] conducted experimental and DFT studies on the pyrolysis of glucose to form HMF, revealing that after the isomerization of glucose to fructose, it similarly undergoes three dehydration reactions. HMF, as a five-membered furan ring, contains active aldehyde and hydroxymethyl groups. It is an important intermediate in the hydrothermal reaction of glucose, exhibiting excellent chemical reactivity. Both subsequent dehydration reactions and polymerization reactions are closely related to HMF.

#### 2.2.1. Mechanistic Analysis of Different Binding Modes of Na Ion with Fructose

**Figure 11:**
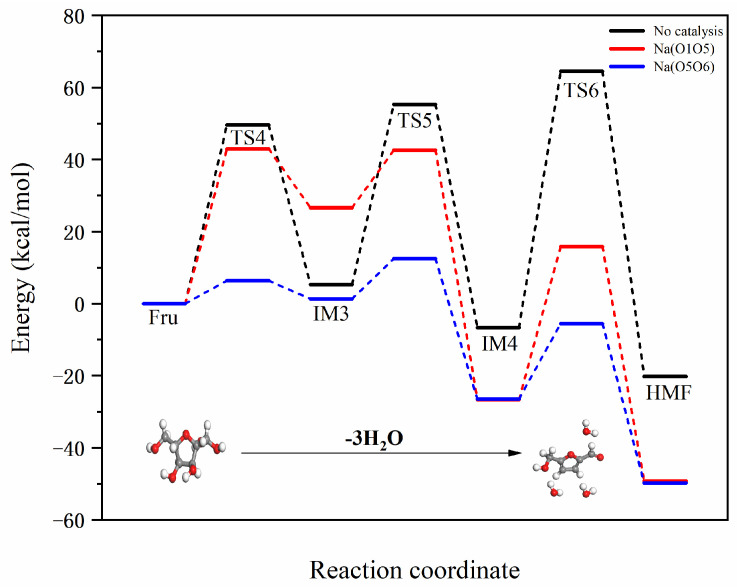
Reaction energy barrier diagrams for two different binding modes of Na^+^ with fructose.

The reaction energy barrier diagram for Na^+^-catalyzed dehydration of fructose to form HMF is shown in Figure 11. Na^+^ binds to the fructose molecule at different positions, primarily facilitating dehydration reactions in three stages. The two different binding modes are uniformly designated as O1O5 and O5O6. In the first dehydration stage, Fru-Na undergoes a dehydration reaction, followed by the transfer of a hydrogen atom from C1 to the hydroxyl group on C2, removing the first water molecule and forming IM3-Na. The reaction energy barriers for the two different positions are 42.97 and 6.40 kcal/mol, respectively. Compared to the non-catalyzed pathway of Fru-IM3, the reaction energy barriers are reduced by 6.65 and 43.22 kcal/mol, respectively. By comparing the reduced reaction energy barriers, it is evident that the catalytic efficiency in this process, from best to worst, is O5O6 > O1O5. This initial dehydration step is the starting point of the entire reaction process, guiding fructose into more complex reaction pathways.

In the second dehydration stage, the hydrogen atom of the hydroxyl group at the C1 position transfers to the hydroxyl group at the C3 position, forming the second water molecule and producing IM4-Na. The reaction energy barriers for the two different positions are 15.98 and 11.17 kcal/mol, respectively. Compared to the non-catalyzed pathway of IM3-IM4, the reaction energy barriers are reduced by 34.00 and 38.81 kcal/mol, respectively. By comparing the reduced reaction energy barriers, it is evident that the catalytic efficiency in this process, from best to worst, is O5O6 > O1O5. This step marks the fructose molecule’s gradual progression towards a highly dehydrated state, laying the foundation for the eventual formation of HMF. The final step involves the transfer of the hydrogen atom at the C5 position to the hydroxyl group at the C4 position, forming the third water molecule and yielding HMF-Na. This is not only the terminal step in the conversion of fructose to HMF but also a critical step in forming the final product. The reaction energy barriers for the two different positions are 42.40 and 20.90 kcal/mol, respectively. Compared to the non-catalyzed pathway of IM4-HMF, the reaction energy barriers are reduced by 28.72 and 50.26 kcal/mol, respectively. By comparing the reduced reaction energy barriers, it is clear that the catalytic efficiency in this process, from best to worst, is O5O6 > O1O5. 

#### 2.2.2. Mechanistic Analysis of Different Binding Modes of K Ion with Fructose

**Figure 12:**
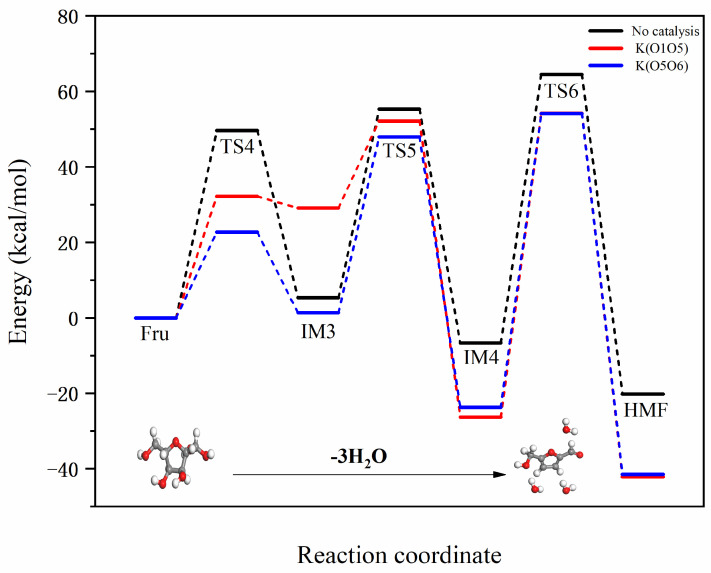
Reaction energy barrier diagrams for two different binding modes of K^+^ with fructose.

The reaction energy barrier diagram for K^+^-catalyzed dehydration of fructose to form HMF is shown in Figure 12. K^+^ binds to the fructose molecule at different positions, facilitating dehydration reactions in three stages. The two different binding modes are uniformly designated as O1O5 and O5O6. In the first dehydration stage, Fru-K undergoes a dehydration reaction, followed by the transfer of a hydrogen atom from C1 to the hydroxyl group on C2, removing the first water molecule and forming IM3-K. The reaction energy barriers for the two different positions are 32.18 and 22.70 kcal/mol, respectively. Compared to the non-catalyzed pathway of Fru-IM3, the reaction energy barriers are reduced by 17.44 and 26.92 kcal/mol, respectively. By comparing the reduced reaction energy barriers, it is evident that the catalytic efficiency in this process, from best to worst, is O5O6 > O1O5.

In the second dehydration stage, the hydrogen atom of the hydroxyl group at the C1 position transfers to the hydroxyl group at the C3 position, forming the second water molecule and producing IM4-K. The reaction energy barriers for the two different positions are 23.06 and 46.59 kcal/mol, respectively. Compared to the non-catalyzed pathway of IM3-IM4, the reaction energy barriers are reduced by 26.91 and 3.38 kcal/mol, respectively. The catalytic efficiency at the O5O6 position is significantly better than at the O1O5 position. By comparing the reduced reaction energy barriers, it is evident that the catalytic efficiency in this process, from best to worst, is O5O6 > O1O5. In the final step, the hydrogen atom at the C5 position transfers to the hydroxyl group at the C4 position, forming the third water molecule and yielding HMF-K. The reaction energy barriers for the two different positions are 80.52 and 77.87 kcal/mol, respectively. Compared to the non-catalyzed pathway of IM5-IM6, the reaction energy barriers are increased by 9.38 and 6.74 kcal/mol, respectively. This final dehydration step indicates that the K^+^ ion does not catalyze this process effectively. Overall, considering the reduction in reaction energy barriers, the catalytic efficiency order from best to worst is O1O5 > O5O6. 

In the study of the mechanism by which Na^+^ and K^+^ ions catalyze the conversion of fructose to HMF, the introduction of potassium or sodium ions can create specific dehydration pathways, resulting in intermediates that favor the formation of HMF. This effect is achieved through the coordination capabilities of these ions, which influence the positions of the aldehyde and hydroxyl groups during the reaction, thereby controlling the sites and extent of dehydration. Potassium or sodium ions may form complexes with water molecules involved in the reaction, which helps accelerate the dehydration process. This increase in catalytic activity can lead to higher reaction rates and greater yields of HMF. The introduction of different ions can impact the competition among various reaction channels, thereby adjusting the product distribution. By controlling the conditions under which complexation reactions occur, it is possible to selectively promote or inhibit specific reaction pathways to achieve higher purity HMF. The presence of sodium or potassium ions can stabilize reaction intermediates by forming stable complexes, preventing further reactions or side reactions.

## 3. Materials and Methods

All calculations in this study were performed using the DMol^3^ module in Materials Studio. The initial configurations of the reactants, intermediates, and final products involved in the initial reactions of cellulose hydrothermal degradation were optimized using the DMol3 module to obtain the most stable geometric structures. Subsequently, transition state searches were conducted for the relevant reactants and their corresponding products. Dispersion-corrected density functional theory (DFT-D) [23], which has been proven to accurately describe non-covalent interactions in aqueous environments, was employed. The solvent type is set as water, and the temperature is set to 475K. Considering the significant changes in the conductivity and dielectric constant of water in the subcritical environment of HTC, these factors were also incorporated into the calculations. All calculations in this study employed the Conductor-like Screening Model (COSMO), a continuum solvation model widely used for computing the chemical properties of organic molecules in water or other solvents. The Perdew–Burke–Ernzerhof (PBE) functional based on the generalized gradient approximation (GGA) method is used to describe the exchange-correlation potential in the aqueous environment. All atoms are treated using all-electron pseudo-potentials, and the valence electron wave functions are expanded using the double numerical plus polarization (DNP) [24] basis set. The structural optimization calculations employed the following convergence criteria: the total energy change of the system during the optimization process is less than 1.0 × 10^−5^ Ha, the maximum change in atomic positions is less than 0.005 Å, and the maximum gradient of the total energy with respect to atomic positions is less than 0.002 Ha/Å [25]. Full geometric optimization was performed for the reactants, all products, and intermediates, and the optimized structures were verified through vibrational analysis, with no imaginary frequencies observed. The transition states (TS) for all reaction steps were located using the complete linear/quadratic synchronous transit (LST/QST) method, and their validity was confirmed by the presence of a single imaginary frequency.

**Figure 13:**
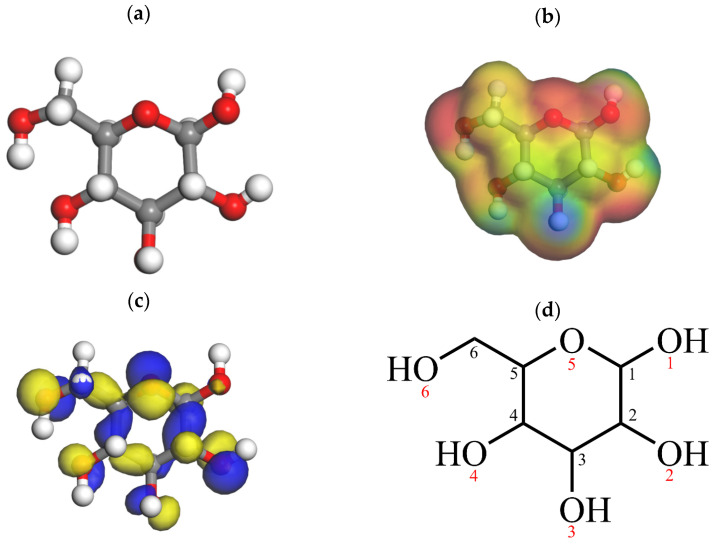
(**a**) Glucose molecular model; (**b**) electrostatic potential map of glucose molecule; (**c**) HOMO map of glucose molecule; and (**d**) spatial configuration of glucose molecule. (Black represents the order of carbon atoms, and red represents the order of oxygen atoms).

Sugars are transformed into various important intermediate products during hydrothermal reactions. Therefore, this study selected glucose as the model compound for the hydrothermal reaction. First, geometry optimization and frequency calculations were performed on the glucose molecule, and the most stable conformation with the lowest energy was chosen as the initial reactant. Figure 13 shows the optimized molecular model of glucose, and Table 1 lists the lengths of the C-O and C-C bonds in the optimized glucose molecule. Next, the potentially active sites in the glucose molecule were identified for the analysis of the reaction. By calculating the possible transition states and intermediates for each step, the hydrothermal reaction system was further elaborated. Finally, K and Na ions were introduced into the system to investigate their catalytic effects on the key reaction steps during the hydrothermal process. Based on the Fukui function calculation results, O5 and O6 exhibit higher nucleophilicity, primarily due to the distribution characteristics of the surrounding electron density. Specifically, O6, located at the end of the glucose molecule chain, has a dense electron cloud around it, making it prone to form electrostatic interactions with positively charged Na^+^ and K^+^. Similarly, O5, due to its position and molecular configuration, tends to undergo ring-opening reactions first, also displaying higher reactivity. This is consistent with previous hydrothermal carbonization studies. O5 and O6, due to their locations, are more likely to form stable coordination structures with larger ions such as Na^+^ and K^+^.

The Fukui function analysis of glucose molecules can be used to assess their local reactivity and identify the most reactive atoms, as well as those most likely to undergo electrophilic or nucleophilic reactions. In Fukui function analysis in Table 2, a larger *f*− value indicates a greater susceptibility to electrophilic attack, while a larger *f*+ value indicates a greater susceptibility to nucleophilic attack. By analyzing the Fukui functions, the relative ease of electrophilic and nucleophilic reactions at different sites of the glucose molecule can be compared. The *f*− value for the oxygen atom at position 5 is 0.131, indicating that this atom is more likely to be attacked by electrophilic reagents and is the most reactive site in the reaction system. Therefore, it can be inferred that the six-membered ring of the glucose molecule is likely to undergo ring-opening reactions first. This prediction is consistent with research findings on glucose conversion and also aligns with the results shown in the electrostatic potential map and HOMO diagram of the glucose molecule (Figure 13).

## 4. Conclusions

During the Na^+^-catalyzed isomerization of glucose to fructose, considering the reaction energy barriers at the three stages and the overall process, the catalytic efficiency of Na^+^ at different positions is evaluated as follows: O1O2 > O4O6 > O3O4 > O5O6. The best catalytic effect is achieved when Na^+^ forms a coordination bond at the O1O2 position. The further Na^+^ is from the hydrogen atom involved in the reaction, the poorer the catalytic effect. In the case of K^+^-catalyzed isomerization of glucose to fructose, the overall evaluation of the catalytic efficiency of K^+^ at different positions is O3O4 > O5O6. In the TS1 reaction stage, the energy barrier reduction for Na-O1O2 is 37.16 kcal/mol, making Na-O1O2 the most effective overall catalytic position compared to other locations. In the TS2 reaction stage, the energy barrier reduction for K-O3O4 is 54.35 kcal/mol, indicating that K-O3O4 has the best overall catalytic effect at this stage. In the TS3 stage, the energy barrier reduction for Na-O3O4 is 29.11 kcal/mol, while for K-O5O6 it is 31.50 kcal/mol. Therefore, in the third stage, K-O5O6 is the most effective catalytic position. During the Na^+^-catalyzed dehydration of fructose to form HMF, considering the energy barriers at the three dehydration stages and the overall process, the catalytic efficiency of Na^+^ at different positions is evaluated as follows: O5O6 > O1O5. The best catalytic effect is achieved when Na^+^ forms a coordination bond at the O5O6 position. In the K^+^-catalyzed dehydration of fructose to form HMF, the overall evaluation of the catalytic efficiency of K^+^ at different positions is O1O5 > O5O6. During the isomerization of glucose molecules to fructose, a comprehensive evaluation of the overall catalytic effect of different ions at different stages showed that Na-O1O2 had excellent catalytic effects in all three stages. During the dehydration of fructose molecules to form HMF, a comprehensive evaluation of the overall catalytic effect of different ions at different stages showed that Na-O5O6 had excellent catalytic effects in all three stages. From the above results, it can be seen that the catalytic effect of Na^+^ is better than that of K^+^ during the hydrothermal carbonization process.

## Figures and Tables

**Table 1:** C-C and C-O bond lengths of glucose molecules.

Bond	Length (Å)	Bond	Length (Å)
C1-O1	1.403	C1-C2	1.533
C2-O2	1.432	C2-C3	1.525
C3-O3	1.437	C3-C4	1.531
C4-O4	1.440	C4-C5	1.534
C5-O5	1.441	C5-C6	1.530
C6-O6	1.434	C1-O5	1.441

**Table 2:** Fukui function values of glucose molecules.

Atom	*f*−	*f+*	Atom	*f*−	*f+*
O1	0.026	0.009	C1	−0.001	0.008
O2	0.111	0.008	C2	−0.006	0.007
O3	0.066	−0.040	C3	−0.007	0.002
O4	0.055	−0.052	C4	−0.001	0.010
O5	0.131	−0.006	C5	−0.015	0.041
O6	0.128	−0.006	C6	−0.006	0.016

## Data Availability

The original contributions presented in the study are included in the article and Supplementary Material, further inquiries can be directed to the corresponding author.

## Supplementary Materials

The following supporting information can be downloaded at: https://www.mdpi.com/article/10.3390/molecules29204849/s1, Figure S1: Electrostatic Potential Maps of HMF; Table S1: Transition state imaginary frequencies of glucose to fructose at positions 12 and 34; Table S2: Fukui Function Values for C and O Bonds in HMF Molecule; Table S3: Fukui function values of glucose molecules; Table S4: Atomic coordinate system of HMF; Table S5: Atomic coordinate system of Glu; Table S6: Atomic coordinate system of Fru; Table S7: Atomic coordinate system of IM1; Table S8: Atomic coordinate system of IM2.

## References

[1] Zhou Y., Zhang Z., Zhang Y., Wang Y., Yu Y., Ji F., Ahmad R., Dong R. (2016). A Comprehensive Review on Densified Solid Biofuel Industry in China. Renew. Sust. Energ. Rev..

[2] Yuan H., Zhu N., Song F. (2011). Dewaterability Characteristics of Sludge Conditioned with Surfactants Pretreatment by Electrolysis. Bioresour. Technol..

[3] Watson J., Wang T., Si B., Chen W.T., Aierzhati A., Zhang Y. (2020). Valorization of Hydrothermal Liquefaction Aqueous Phase: Pathways towards Commercial Viability. Prog. Energ. Combust..

[4] He C., Chen C., Giannis A., Yang Y., Wang J.Y. (2014). Hydrothermal Gasification of Sewage Sludge and Model Compounds for Renewable Hydrogen Production: A Review. Renew. Sust. Energ. Rev..

[5] Peng C., Zhai Y., Zhu Y., Xu B., Wang T., Li C., Zeng G. (2016). Production of Char from Sewage Sludge Employing Hydrothermal Carbonization: Char Properties, Combustion Behavior and Thermal Characteristics. Fuel.

[6] Tang Z., Boer D., Syariati A., Enache M., Rudolf P., Heeres H.J., Pescarmona P.P. (2019). Base-Free Conversion of Glycerol to Methyl Lactate Using a Multifunctional Catalytic System Consisting of Au-Pd Nanoparticles on Carbon Nanotubes and Sn-MCM-41-XS. Green. Chem..

[7] Hu B., Lu Q., Jiang X., Dong X., Cui M., Dong C., Yang Y. (2018). Pyrolysis Mechanism of Glucose and Mannose: The Formation of 5-Hydroxymethyl Furfural and Furfural. J. Energy Chem..

[8] Weiqi W., Shubin W. (2017). Experimental and Kinetic Study of Glucose Conversion to Levulinic Acid Catalyzed by Synergy of Lewis and Brønsted Acids. Chem. Eng. J..

[9] Zhang T., Wei H., Xiao H., Li W., Jin Y., Wei W., Wu S. (2020). Advance in Constructing Acid Catalyst-Solvent Combinations for Efficient Transformation of Glucose into 5-Hydroxymethylfurfural. Mol. Catal..

[10] Arora J., Ansari K., Chew J., Dauenhauer P., Mushrif S. (2019). Unravelling the Catalytic Influence of Naturally Occurring Salts on Biomass Pyrolysis Chemistry Using Glucose as a Model Compound: A Combined Experimental and DFT Study. Catal. Sci. Technol..

[11] Guo W., Zuo M., Zhao J., Li C., Xu Q., Xu C., Wu H., Sun Z., Chu W. (2020). Novel Brønsted–Lewis Acidic Di-Cationic Ionic Liquid for Efficient Conversion Carbohydrate to Platform Compound. Cellulose.

[12] Guo S., Xiao W., Zhao D., Liu Z., Liu L., Xu D., Li X., Li G. (2023). Water-Catalyzed Conversion of Glucose to Small Molecules during Hydrothermal Carbonization: A Density Functional Theory Study. Sustain. Energ. Fuels.

[13] Abdullayev Y., Ahmadov O., Valadova G., Karimli A., Autschbach J. (2021). Unveiling the Catalytic Effects of Brønsted Acidic Ionic Liquid on Quantitative α-Glucose Conversion to 5-HMF: Experimental and Computational Studies. Renew. Energ..

[14] Tian G., Tong X., Cheng Y., Xue S. (2013). Tin-Catalyzed Efficient Conversion of Carbohydrates for the Production of 5-Hydroxymethylfurfural in the Presence of Quaternary Ammonium Salts. Carbohyd Res..

[15] Moliner M., Román-Leshkov Y., Davis M. (2010). Tin-Containing Zeolites Are Highly Active Catalysts for the Isomerization of Glucose in Water. Proc. Natl. Acid. Sci. USA.

[16] Zhang J., Cao Y., Li H., Ma X. (2014). Kinetic Studies on Chromium-Catalyzed Conversion of Glucose into 5-Hydroxymethylfurfural in Alkylimidazolium Chloride Ionic Liquid. Chem. Eng. J..

[17] Pyo S., Glaser S., Rehnberg N., Hatti-Kaul R. (2020). Clean Production of Levulinic Acid from Fructose and Glucose in Salt Water by Heterogeneous Catalytic Dehydration. ACS Omega.

[18] Li J., Li J., Zhang D., Liu C. (2015). Theoretical Elucidation of Glucose Dehydration to 5-Hydroxymethylfurfural Catalyzed by a SO3H-Functionalized Ionic Liquid. J. Phys. Chem. B.

[19] Parveen F., Upadhyayula S. (2017). Efficient Conversion of Glucose to HMF Using Organocatalysts with Dual Acidic and Basic Functionalities-A Mechanistic and Experimental Study. Fuel Process Technol..

[20] Yang Y., Liu W., Wang N., Wang H., Song Z., Li W. (2015). Effect of Organic Solvent and Broønsted Acid on 5-Hydroxymethylfurfural Preparation from Glucose over CrCl3. RSC Adv..

[21] Guo S., Liu Q., Zhao D., Liu Z., Chen K., Li X., Li G. (2023). Density Functional Theory Study of Acid-Catalyzed Conversion of Glucose to Hydrochar Precursors under Hydrothermal Conditions. Energy.

[22] Kang X., You Z., Huang Y. (2024). Multi-catalytic active site biochar-based catalysts for glucose isomerized to fructose: Experiments and density functional theory study. Adv. Compos. Hybrid. Mater..

[23] Grimme S., Antony J., Ehrlich S., Krieg H. (2010). A Consistent and Accurate Ab Initio Parametrization of Density Functional Dispersion Correction (DFT-D) for the 94 Elements H-Pu. J. Chem. Phys..

[24] Kryachko E., Ludeña E. (2014). Density Functional Theory: Foundations Reviewed. Phys. Rep..

[25] Ferrighi L., Pan Y., Grönbeck H., Hammer B. (2012). Study of Alkylthiolate Self-Assembled Monolayers on Au(111) Using a Semilocal Meta-GGA Density Functional. J. Phys. Chem. C.

