# Supplementary material for: Density Functional Theory Study on Na+ and K+ Catalysis in the Transformation of Glucose to Fructose and HMF in Hydrothermal Environments
Source: Molecules. 2024 Oct 13;29(20):4849. doi: 10.3390/molecules29204849 (PMC11510219; doi:10.3390/molecules29204849)
Supplement: Supplementary file 1 [file molecules-29-04849-s001.zip › molecules-3211943-supplementary.pdf]

Supplementary Materials

Density functional theory study on Na^+ and K^+ catalysis in the transformation of glucose to fructose and HMF in hydrothermal environments

Long Gao[1], Qihao Chen[1], Yanhong Wang[1,*], Deyong Che[1], Baizhong Sun[1] and Shuai Guo[1,]**

[1.] School of Energy and Power Engineering, Northeast Electric Power University, Jilin 132012, China; gaolong0607@163.com (L.G.); 19861834570@163.com (Q.C.); wangyanhong1985@126.com (Y.W.); chedeyong163@163.com (D.C.); sunbaizhong@126.com (B.S.); guoshuaidq@126.com (S.G.)

* Correspondence: 20162710@neepu.edu.cn (Y.W.); 20162704@neepu.edu.cn (S.G.)

Glu	Glu-12-Na	Glu-34-Na
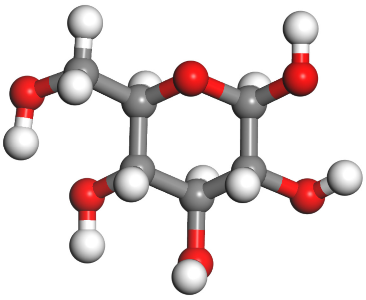	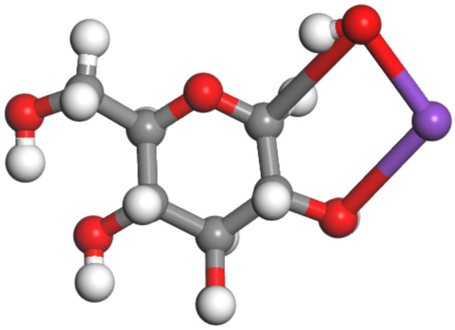	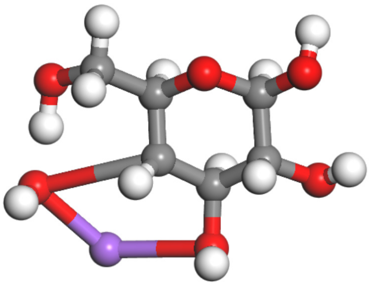
IM2	IM2-12-Na	IM2-34-Na
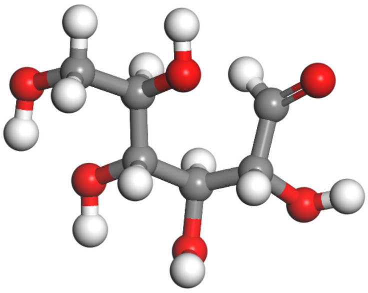	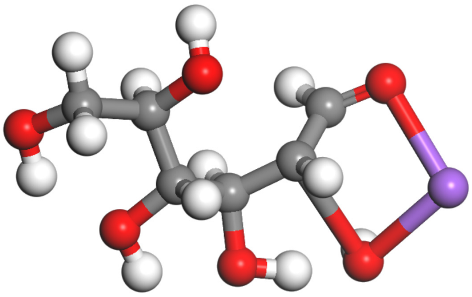	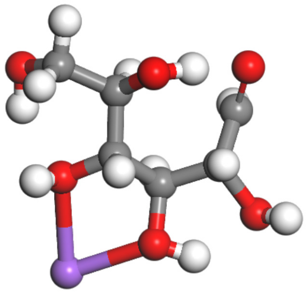
IM3	IM3-12-Na	IM3-34-Na
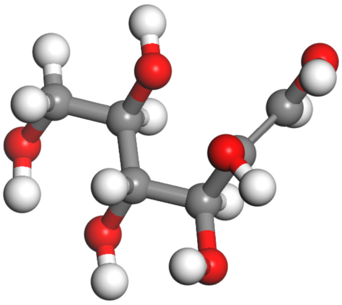	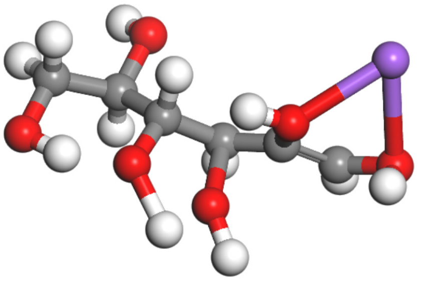	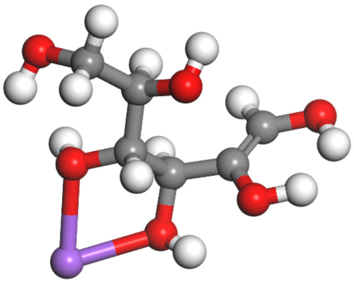
Fru	Fru-12-Na	IM2-34-Na
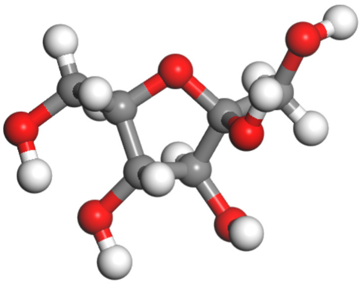	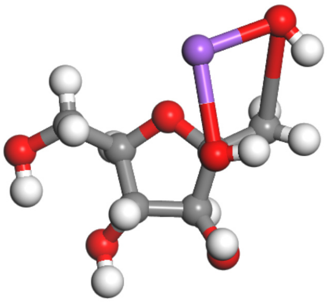	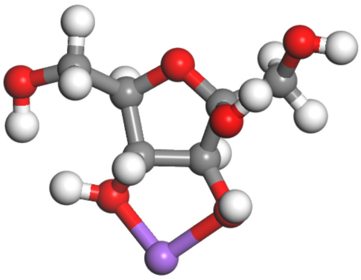
Glu	Glu-56-Na	Glu-46-Na

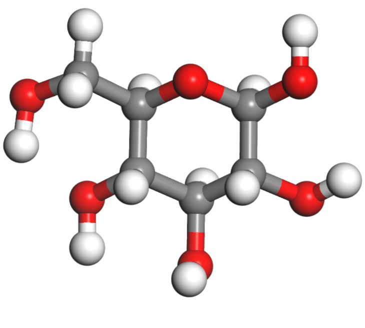

IM2

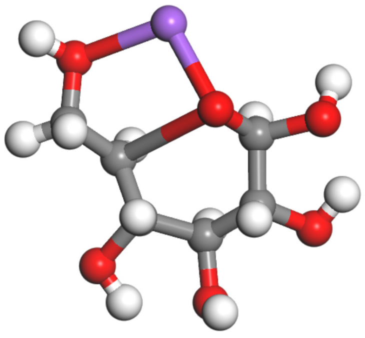

IM2-56-Na

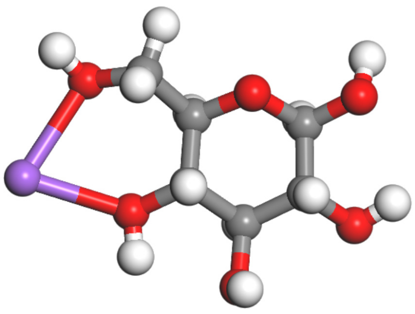

IM2-46-Na

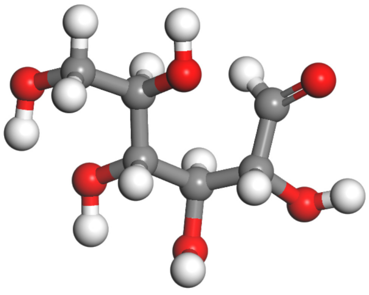

IM3

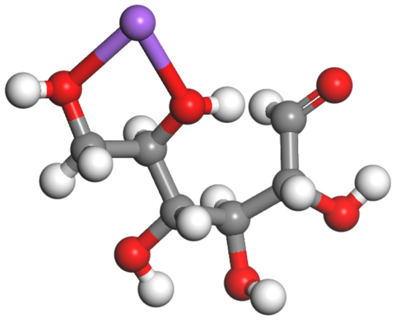

IM3-56-Na

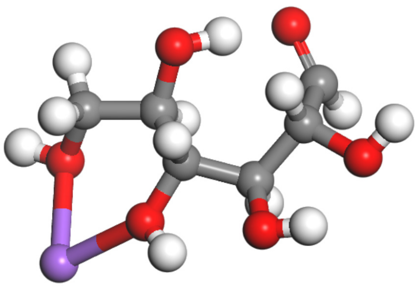

IM3-46-Na

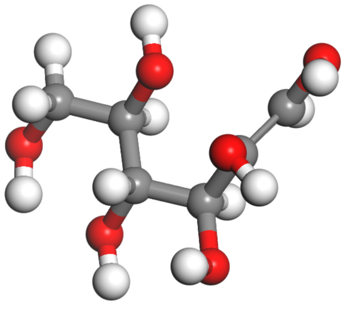

Fru

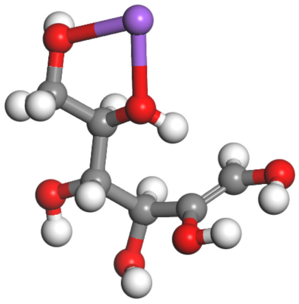

Fru-56-Na

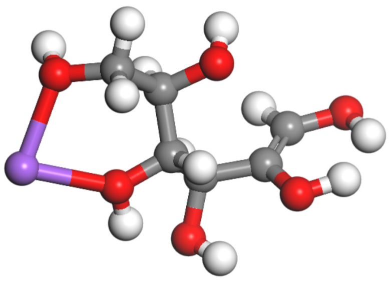

IM2-46-Na

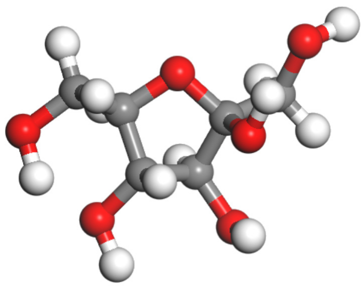

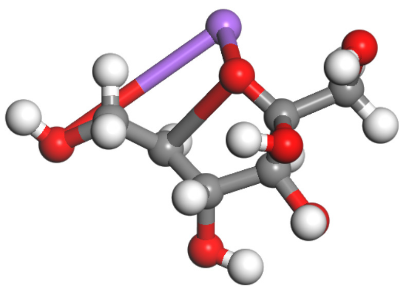

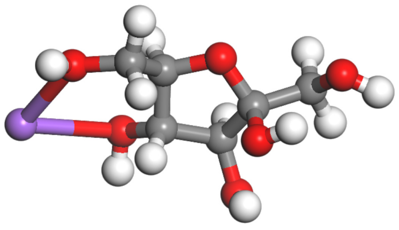

Glu	Glu-56-K	Glu-34-K
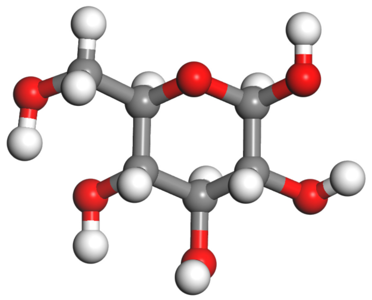	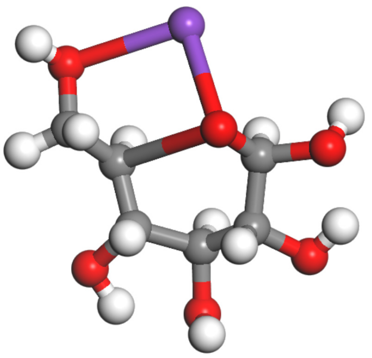	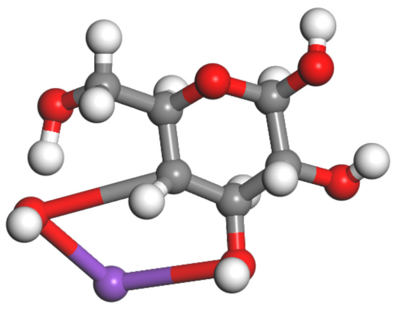
IM2	IM2-56-K	IM2-34-K
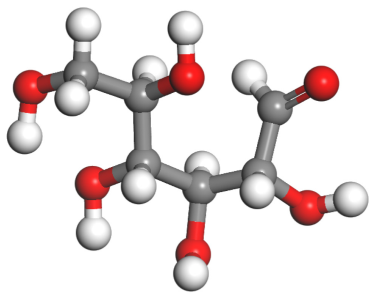	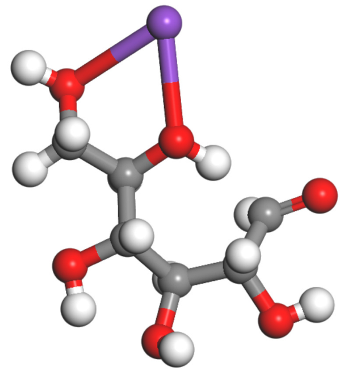	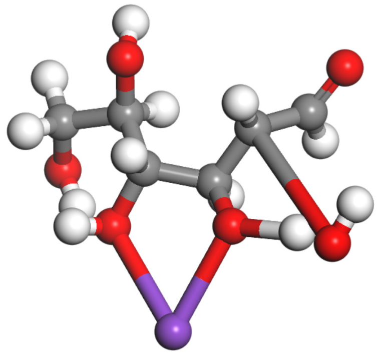
IM3	IM3-56-K	IM3-34-K
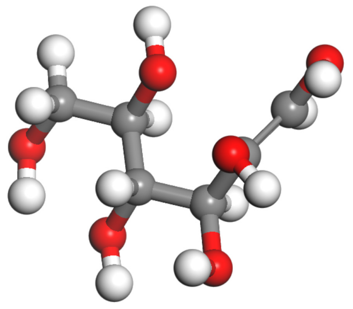	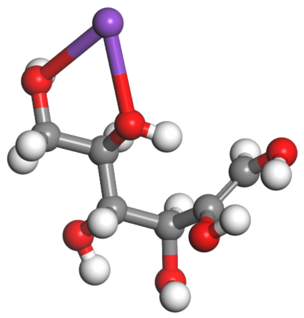	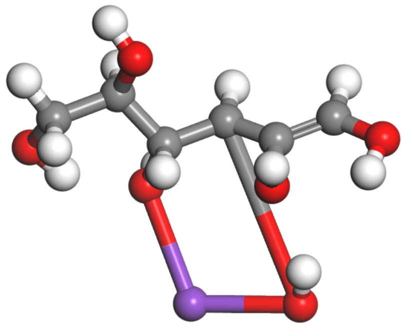
Fru	Fru-56-K	IM2-34-K
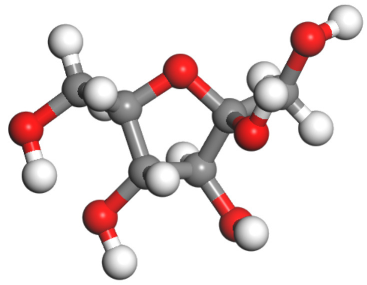	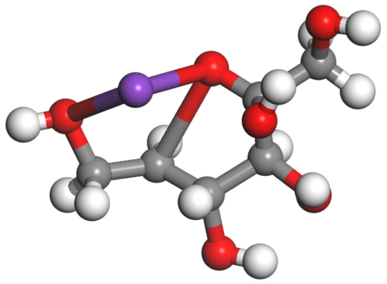	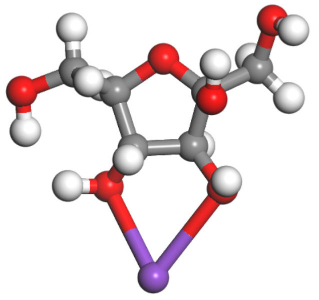

Table S1. Transition state imaginary frequencies of glucose to fructose at positions 12 and 34.

Transition state	Imaginary frequency	Transition state	Imaginary frequency	Transition state	Imaginary frequency
TS1	-1528.92	TS1-O1O2	-124.05	TS1-O3O4	-150.27
TS2	-2144.29	TS2-O1O2	-326.58	TS2-O3O4	-168.77
TS3	-1429.62	TS3-O1O2	-315.90	TS3-O3O4	-175.36

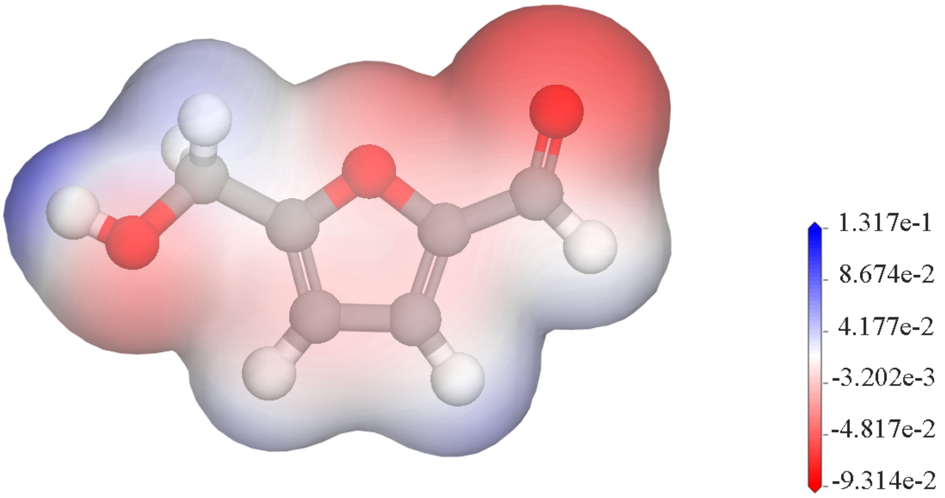

Figure S1. Electrostatic Potential Maps of HMF.

Table S2. Fukui Function Values for C and O Bonds in HMF Molecule.

**Atom**	***f-***	***f+***	***f0***	**Atom**	***f-***	***f+***	***f0***
C1	0.188	0.066	0.127	C6	-0.001	0.004	0.034
C2	0.037	0.084	0.061	O1	0.161	0.206	0.183
C3	0.127	0.066	0.066	O5	0.051	0.032	0.042
C4	0.042	0.077	0.077	O6	0.009	0.061	0.035
C5	0.101	0.078	0.078				

Table S3. Fukui function values of glucose molecules.

**Atom**	***f*-**	***f*+**	**Atom**	***f*-**	***f*+**
O1	0.026	0.009	C1	-0.001	0.008
O2	0.111	0.008	C2	-0.006	0.007
O3	0.066	-0.040	C3	-0.007	0.002
O4	0.055	-0.052	C4	-0.001	0.010
O5	0.131	-0.006	C5	-0.015	0.041
O6	0.128	-0.006	C6	-0.006	0.016

Table S4. Atomic coordinate system of HMF.

coordinate system	atomic coordinates		
	x	y	z
C	-1.25163	1.12548	-0.29474
C	1.12908	1.98147	-0.92584
C	1.03643	4.64366	-0.83027
C	-1.41182	5.29189	-0.15297
O	-2.82695	3.09946	0.17422
C	-2.69152	7.65095	0.17427
C	-2.32073	-1.47774	-0.02948
O	-0.43241	-3.36875	-0.34444
O	-1.65196	9.75914	0.04606
H	2.75091	0.81058	-1.38620
H	2.53828	5.97178	-1.23816
H	-4.75406	7.49737	0.54386
H	-3.74894	-1.80820	-1.51480
H	-3.29757	-1.63973	1.81340
H	0.70645	-3.26214	1.11656

Table S5. Atomic coordinate system of Glu.

coordinate	X	Y	Z
C	-1.41755	-1.47733	-1.01052
C	1.054472	-1.72167	0.486753
C	2.646001	0.69084	0.280743
C	1.074942	3.008215	0.974526
C	-1.40541	2.988954	-0.52861
O	-2.88187	5.020398	0.332763
O	2.509036	5.234248	0.425058
O	4.923782	0.460232	1.740046
O	2.440814	-3.85896	-0.46097
C	-3.16105	-3.76306	-0.69437
O	-2.1924	-5.97632	-1.91347

O	-2.78115	0.686394	-0.08289
H	-0.9708	-1.23444	-3.04624
H	0.555289	-2.03099	2.501951
H	3.295814	0.887058	-1.69317
H	0.571506	2.930945	3.010795
H	-1.00433	3.16171	-2.57899
H	-4.10066	5.437295	-1.00883
H	1.344043	6.65673	0.721426
H	4.415334	0.413059	3.530869
H	4.00887	-3.92653	0.544844
H	-4.99593	-3.33361	-1.58245
H	-3.49105	-4.09948	1.347631
H	-0.43771	-6.1537	-1.29689

Table S6. Atomic coordinate system of Fru

C	-1.47495	-2.04196	-0.94948
C	1.416573	-1.99938	-0.85833
C	2.00587	0.831665	-1.09888
C	-0.13705	1.989151	0.483778
C	-0.75906	4.744225	-0.14862
O	-2.17025	5.837821	1.910315
O	0.577502	1.812168	3.065399
O	4.467131	1.569815	-0.31883
O	2.433208	-3.56346	-2.81642
C	-2.6827	-4.11956	0.645235
O	-2.23367	-6.57528	-0.42182
O	-2.29939	0.423465	-0.05249
H	-2.08043	-2.26641	-2.93936
H	2.096136	-2.65793	1.008836
H	1.788675	1.396431	-3.09604
H	-1.8825	4.809739	-1.90275
H	-0.32705	3.244195	3.880931
H	4.374786	1.645935	1.545496
H	4.259585	-3.68313	-2.48918
H	-4.74899	-3.86297	0.68738
H	-1.96518	-3.99183	2.60852
H	-0.40139	-6.701	-0.72398
H	-1.2853	7.386815	2.417054
H	1.028444	5.771489	-0.43675

Table S7. Atomic coordinate system of IM1

C	-3.79177	0.287431	-0.82023
C	-1.4535	0.611819	0.841606
C	-0.03187	3.130623	0.524242
C	-1.48449	5.500658	1.316722
C	-3.63888	6.274127	-0.4562
O	-5.27567	7.786107	0.19879
O	0.271338	7.590663	1.182696
O	2.303067	2.925468	1.912727
O	0.279651	-1.38962	0.258323
C	-4.88529	-2.40332	-0.70559
O	-3.44642	-4.20874	-2.12032
O	-5.6093	2.054205	0.188442
H	-3.33626	0.747771	-2.81049
H	-2.08185	0.453482	2.839306
H	0.55617	3.312308	-1.46873
H	-2.23511	5.283605	3.254167
H	-3.43788	5.701229	-2.47754
H	-0.65914	9.078036	1.808322
H	1.867095	2.961334	3.724569
H	1.903996	-0.81456	0.990105
H	-6.78981	-2.37035	-1.55734
H	-5.08128	-2.97264	1.305457
H	-1.70863	-4.06454	-1.45833
H	-6.97811	2.20617	-1.06103

Table S8. Atomic coordinate system of IM2

C	-1.79211	-1.90552	-0.20745
C	0.934164	-1.41748	0.687328
C	2.086966	0.982193	-0.50873
C	0.813327	3.369502	0.268849
C	-0.52001	4.848764	-1.30827
O	-1.7799	6.980553	-0.5644
O	0.893509	3.992877	2.856536
O	4.788382	1.009484	0.029505
O	2.531196	-3.54209	0.072414
C	-2.43773	-4.72704	-0.45519
O	-1.24183	-5.88256	-2.5965
O	-3.41521	-0.72558	1.629814
H	-2.07245	-1.02377	-2.08722
H	0.88102	-1.186	2.768792

H	1.961823	0.792297	-2.58077
H	-0.71426	4.451652	-3.32247
H	2.373627	5.085988	3.139375
H	4.94898	1.325136	1.859012
H	4.241868	-2.78815	-0.05445
H	-4.4951	-4.8956	-0.74727
H	-1.95666	-5.72193	1.323843
H	0.578161	-5.55746	-2.3126
H	-5.08	-0.56306	0.821788
H	-1.52776	7.097777	1.288068
